# Referral and Activity in Specialist Psycho‐Oncology Services: A Survey of Provision in England and Scotland

**DOI:** 10.1002/pon.70613

**Published:** 2026-09-18

**Authors:** Anna Lagerdahl, Vanessa Bútorová

**Affiliations:** ^1^ Clinical Health Psychology Great Western Hospitals NHS Foundation Trust Swindon UK; ^2^ School of Psychology and Neuroscience University of Bristol Bristol UK

**Keywords:** cancer, health services research, leadership, oncology, psychological, psycho‐oncology, referral and consultation, workforce

## Abstract

**Background:**

Distress is a common, clinically significant response to cancer, yet specialist psycho‐oncology provision varies across the UK, and national service‐level data on staffing, access and activity are lacking.

**Aims:**

To provide an updated picture of referral, access, workforce and activity of specialist psycho‐oncology services across the UK.

**Methods:**

A cross‐sectional service survey distributed UK‐wide via professional networks used an 18‐item questionnaire capturing staffing, referrals, waiting times, activity and outcome measures for 2022/2023. Activity was expressed per specialist whole‐time equivalent (WTE); reporting followed STROBE.

**Results:**

Twenty‐five psychology‐led NHS psycho‐oncology services participated, all in England and Scotland. All had Level 4 provision but only 40% had Level 3. Each WTE served a mean of 1530 new cancer diagnoses, saw 83 new patients and delivered 446 attended appointments (mean 7 sessions per patient). 8.4% of patients were referred for specialist support, below the ∼25% estimated to need it. Eighty‐three percent of services judged their referrals to under‐represent need. Mean waits were 21 days (urgent) and 3.4 months (routine); services without a dedicated senior leadership post tended to wait longer (median routine 4 vs. 3 months; urgent 30 vs. 10 days).

**Conclusions:**

Specialist psycho‐oncology services in England and Scotland are understaffed, with long waits and inequitable access, even among psychology‐led services. Senior leadership may support shorter waits but must be matched by balanced staffing across Levels 3 and 4. Workforce investment, inclusive referral pathways, a cancer‐specific psychological wellbeing measure, and embedding specialist support where it is absent are needed to deliver equitable care.

## 1 Background

Cancer is one of the most common serious illnesses in the UK, with around 420,000 people diagnosed each year and incidence continuing to rise as the population ages [1, 2]. Alongside its physical burden, a cancer diagnosis can provoke a profound psychological response in a range of areas, including low mood, anxiety, fear of recurrence [3], loss of control and existential struggles [4], to name a few. Psycho‐oncology, the discipline addressing these needs, was named by Jimmie Holland, who argued for integrating mental health support into cancer services and emphasised that distress is a normal, expected reaction to life‐threatening illness rather than a sign of pathology [5]. Normalising distress does not diminish its clinical significance: it is common and often persistent [3]. A recent umbrella review estimated that around a third of people with cancer experience clinically significant depression (33%) or anxiety (31%) [6], with difficulties that can outlast treatment and undermine adherence, quality of life and recovery [7, 8].

Most UK psycho‐oncology services follow the four‐tier stepped‐care model set out by the National Institute for Health and Care Excellence (NICE) in 2004 [9], in which all cancer staff provide psychological support at levels matched to need. Around a quarter of people diagnosed with cancer are estimated to require specialist input—roughly 15% at Level 3 (e.g., counsellors and psychotherapists) and 10% at Level 4 (e.g., clinical and counselling psychologists)—with additional distress in advanced disease [10]. Yet specialist provision rarely matches this modelled need, with persistent under‐resourcing and explicit calls for change [11]. The central planning challenge is therefore need versus provision: a large, well‐evidenced psychological need on one side, and a specialist workforce whose size, configuration and reach remain poorly quantified on the other.

In 2015 the British Psychological Society (BPS) published *Demonstrating Quality and Outcomes in Psycho‐Oncology*, setting out domains (safety, equity, timeliness, effectiveness and efficiency) against which services could be described and benchmarked [12]. A decade on, its referral and activity domains remain only partially characterised nationally. Regional mapping exercises of psycho‐oncology services have repeatedly found understaffing and a shortfall of psychological specialists relative to need [13, 14, 15], and international analyses continue to highlight substantial unmet psychosocial need and comparatively modest investment in the field [16, 17]. There is no up to date national account of how UK psycho‐oncology services are staffed, whom they accept, how long patients wait, and what activity they deliver.

This study had two related purposes. The first was to provide an updated, UK‐wide picture of the referral and activity domains set out in the 2015 BPS framework. The second was to increase understanding of how UK psycho‐oncology services operate: their workforce, referral routes, waiting times and activity, so as to inform both the development of existing services and the case for establishing specialist psychological provision in areas where none currently exist. Because service sizes vary widely, activity is expressed throughout per whole‐time‐equivalent (WTE) specialist psychology staff.

## 2 Methods

### 2.1 Design

A cross‐sectional service survey of specialist psycho‐oncology services was undertaken across the UK, capturing service‐level quantitative data and free‐text responses for the fiscal year 2022/2023 (April 2022–March 2023); completed responses were received from services in England and Scotland only. Reporting adhered to the STROBE guidance as far as applicable to a service‐level survey.

### 2.2 Participants

The survey was circulated across the UK through a national psycho‐oncology leads network and other professional networks and groups, which were asked to disseminate it onward to relevant services; participation was voluntary. Because it was distributed in this cascaded way rather than sent to a defined sampling frame, the number of services reached, eligible or declining is not known. Completed responses were received only from services in England and Scotland; none were returned from Wales or Northern Ireland. For context, cancer care in England is delivered by around 120 NHS provider trusts across 20 Cancer Alliances, and in Scotland by the 14 territorial NHS boards across three regional cancer networks; there is no UK‐wide registry of specialist (Level 3–4) psycho‐oncology services, and regional mapping exercises have found only a few in each area [13, 14, 15]. The responding services should therefore be regarded as a self‐selected sample, probably weighted towards better‐established, psychology‐led services, rather than a comprehensive or representative census. Some services were unable to complete every item on the questionnaire, so the number of contributing services (*N*) is reported for each metric.

### 2.3 Materials

An 18‐item structured questionnaire was developed with input from members of the ‘National Psycho‐Oncology Leads Network’ and was also informed by the BPS Demonstrating Quality and Outcomes in Psycho‐Oncology framework [12]. Items captured the number of new cancer patients registered in the host trust; workforce at Levels 3 and 4 (by Agenda for Change band [18]—the national NHS pay and grading scale, on which band 8b and above denotes a senior psychologist post, with consultant‐level roles typically banded 8c or 8d; substantive or fixed‐term); referral and exclusion criteria; who could refer to the service; number of referrals; waiting times for routine and urgent referrals; numbers of new and follow‐up appointments; inpatient referral acceptance; percentages of ‘cancelled at short notice’ and non‐attended appointments; average number of sessions attended per patient; specialist provision; and outcome measures used. A final item captured whether respondents felt referral numbers reflected need, alongside free‐text contextual comments.

### 2.4 Procedure

The questionnaire and a consent form were emailed to eligible leads, who were invited to participate voluntarily. Completed surveys and consent forms were returned by email to the lead researcher.

### 2.5 Analysis

Quantitative and qualitative responses were analysed separately. Quantitative activity data were analysed descriptively; to enable meaningful comparison across services of markedly different sizes, activity was standardised to the available specialist workforce (Level 3–4) and expressed per WTE (new cancer diagnoses, new patients and attended appointments per WTE) and summarised as the number of contributing services (*N*), mean, median and range. Routine and urgent waiting times were each compared between services with and without a dedicated senior psychological leadership post (a band 8b, 8c or 8d post of at least 0.4 WTE). The association between referral rate and both catchment size (new diagnoses) and specialist staffing (WTE per new diagnosis) was examined using Spearman's rank correlation; given the small number of services, all other comparisons are descriptive. Free‐text responses concerning referral criteria, accepted referrers and outcome measures were examined using content analysis to identify and quantify recurring categories. Missing items were handled by pairwise exclusion, and the number of contributing services is reported for each metric.

### 2.6 Ethics

This service evaluation was approved by the University of Bristol School of Psychological Science Student Research Ethics Committee (reference 15696; 29 June 2023) and conducted in accordance with the principles of the Declaration of Helsinki. Participants were service leads, who provided informed written consent. Only anonymised, service‐level data were collected, no patient‐identifiable information, and all data are reported at service level.

## 3 Results

### 3.1 Staffing and Service Configuration

Twenty‐five self‐selected NHS specialist psycho‐oncology services took part, all psychology‐led. Participating services spanned England (*N* = 22) and Scotland (*N* = 3) and were predominantly employed by acute trusts (hospitals providing physical cancer care; *N* = 21) with a couple employed by mental health trusts, and two more through service‐level agreements (formal inter‐trust arrangements) between a mental health trust and an acute trust. Service size varied widely, with new cancer diagnoses ranging from 940 to 11,104 in 2022/2023 and specialist team sizes from 0.6 to 11.66 WTE. All participating services had Level 4 provision, but only 40% had any Level 3 provision—a higher proportion of staffing at Level 4 than Level 3, and an inversion of the stepped‐care model's expectation that the lower specialist tier carries the greater volume. Staff spanned Agenda for Change bands 6 to 8d, with band 8a the most frequently reported (Table 1).

**TABLE 1:** Workforce configuration of participating NHS specialist psycho‐oncology services in 2022/2023.

Workforce indicator	Value
Services with level 4 provision	100%
Services with level 3 provision	40%
Range of specialist team size (WTE)	0.6–11.66
Most frequently reported band	8a

*Note:* Staff spanned Agenda for Change bands 6 to 8d.

Abbreviation: WTE, whole‐time equivalent.

### 3.2 Referral Routes and Criteria

Referral criteria were broadly similar across services (*N* = 25): most services accepted anyone under the care of the trust's cancer team, whose distress related primarily to the cancer diagnosis, its treatment or post‐treatment adjustment. However, the scope of referrals accepted varied. Most services (74%) accepted inpatient referrals but only 8% reported offering support to family members or carers. Referrals were accepted from any healthcare professional within the hospital multidisciplinary team (MDT), with around a third (32%) of participating services also accepting GP referrals, and few (12%) accepting self‐referrals.

### 3.3 Referrals Relative to Need

Across 18 services, a mean of 8.4% of new cancer patients (median 8.0%; range 2.4%–18.8%) were referred to psycho‐oncology, well below the approximately 25% estimated by NICE to require specialist psychological support [10]. Consistent with this, 83% of services judged their referral numbers to under‐represent the true need for specialist psychological support; none considered them an over‐representation. Referral rate was not associated with catchment size (Spearman *ρ* = −0.14, *n* = 18) but was strongly associated with specialist staffing relative to population (WTE per new diagnosis; *ρ* = 0.74, *p* < 0.001), consistent with under‐referral reflecting limited capacity rather than lower demand.

### 3.4 Service Activity per WTE

To make activity comparable across services of very different sizes, workload was expressed per specialist WTE (Levels 3 and 4; Table 2). Each WTE parallelled a mean of 1530 new cancer patients registered in the host service in 2022/2023 (median 1167; range 476–3542), saw a mean of 83 new patients that year (median 79; range 31–142), and delivered a mean of 446 attended appointments (median 460; range 188–770). Patients attended a mean of 7 sessions each (range 4–10), although the number of appointments per patient was described as varying considerably within individual services, depending on patient need.

**TABLE 2:** Service activity per whole‐time‐equivalent (WTE) specialist (Levels 3 and 4) in 2022/2023.

Activity metric	*N*	Mean	Median	Range
New cancer diagnoses per WTE	19	1530	1167	476–3542
New patients seen per WTE	20	83	79	31–142
Attended appointments per WTE	19	446	460	188–770
Sessions per patient	21	7	—	4–10

*Note:* New‐diagnosis data include substantive and fixed‐term posts; the percentage‐referred denominator (see text) varied in whether non‐melanoma skin cancers were included.

Abbreviations: *N*, number of contributing services; WTE, whole‐time equivalent.

### 3.5 Waiting Times and Non‐Attendance

Waiting times were long and variable (Table 3). Urgent referrals waited a mean of 21 days (median 12; range same‐day to 77 days), a considerable wait for referrals designated urgent, and routine referrals waited a mean of 3.4 months (median 3.0; range 0.5–9). The four services without a dedicated senior leadership post tended to wait longer than those with one: median routine waits were 4.25 versus 3.0 months (mean 4.4 vs. 3.2; *n* = 4 vs. 19) and median urgent waits 30 versus 10 days (mean 39 vs. 18; *n* = 3 vs. 17). A mean of 19.9% of appointments were not attended or cancelled, comprising a mean ‘unable‐to‐attend’ (UTA) rate of 12.9% and a ‘did‐not‐attend’ (DNA) rate of 8.1%, with some variability in how trusts recorded UTAs.

**TABLE 3:** Waiting times for urgent and routine referrals in 2022/2023.

Referral type	*N*	Mean	Median	Range
Urgent referrals (days)	20	21 (days)	12	Same‐day–77
Routine referrals (months)	23	3.4 (months)	3.0	0.5–9

*Note:* Wait by leadership (dedicated senior leadership post vs. none; see Methods): routine median 3.0 versus 4.25 months (mean 3.2 vs. 4.4), *n* = 19 versus 4; urgent median 10 versus 30 days (mean 18 vs. 39), *n* = 17 versus 3. Non‐attendance (18 services): combined DNA/UTA 19.9% (UTA 12.9%; DNA 8.1%).

Abbreviations: DNA, did not attend; *N*, number of contributing services; UTA, unable to attend.

### 3.6 Specialist Provision

The majority of participating services (15 of 25) had no specialist provision for particular tumour sites or patient groups. Where present, provision spanned teenagers and young adults, haematology and stem‐cell transplant, palliative care, head and neck, risk‐reducing/symmetrisation breast surgery, familial ovarian cancer, psychosexual therapy, cancer rehabilitation, cancer pain and endocrine treatment.

### 3.7 Outcome Measures

Most services used at least one formal outcome measure, but choice varied widely (Table 4). The PHQ and GAD were the most common, followed by the HADS, CORE‐10 and FACT‐G. Only a minority used a validated quality‐of‐life (QoL) measure, and services often used different instruments, or different versions of the same instrument.

**TABLE 4:** Most frequently reported outcome measures in 2022/2023.

Outcome measure	Services using (*n*)
PHQ (patient health questionnaire)	19
GAD (generalised anxiety disorder scale)	18
HADS (hospital anxiety and depression scale)	5
CORE‐10 (clinical outcomes in routine Evaluation–10)	5
FACT‐G (functional assessment of cancer therapy–General)	5
WEMWBS (warwick–Edinburgh mental wellbeing scale)	3
IES (impact of event scale)	3

*Note:* Services typically reported more than one measure. Other measures reported included the Distress Thermometer, ORS, IPOS, EORTC QLQ‐C30, Mini‐MAC, Appearance Anxiety Inventory, Flourishing Scale and patient feedback.

## 4 Discussion

This survey provides one of the most detailed service‐level accounts to date of specialist psycho‐oncology provision in England and Scotland, describing referral and activity data a decade after the BPS [12] first set out a framework for developing quality and outcomes in psycho‐oncology services. Three key findings stand out: services are markedly understaffed relative to need, patients face long and variable waits, and access is inequitable and inconsistently governed, with the important caveat that all participating services have psychological provision.

Expressed per specialist WTE, the workload is considerable. On average, each WTE corresponded to roughly 1530 new cancer patients in its host service per year; on the NICE [9] estimate that 25% require specialist input, that would imply about 383 new patients per WTE each year. In reality, each WTE saw on average around 83 new patients per year, while delivering 446 attended appointments. Patients seen for therapy received a mean of 7 sessions, depending on individual need. Although no statutory caseload standard exists, the BPS suggests a maximum of 25 active patients per practitioner to maintain quality of care [19]. Compared with general psychological services, which receive proportionally more referrals and typically provide more sessions per patient [20], the very low referral rate (8.4%) combined with long urgent waits points to systemic understaffing rather than inefficiency: the workload appears technically manageable only because most patients in need are never referred. A mean urgent wait of 21 days is considerable for referrals designated urgent, and the concentration of demand on scarce specialist practitioners heightens the risk of burnout in an already pressured workforce [21, 22, 23]. These findings echo regional UK mapping exercises reporting understaffing and a shortfall of specialists relative to need [13, 14, 15]. To our knowledge, no comparable national service‐level surveys have been conducted in the UK or internationally, making these data valuable but limiting direct comparison.

The gap between an 8.4% referral rate and the approximately 25% estimated need, corroborated by 83% of services judging their referrals to under‐represent need, has major implications for equity and access [10]. Under‐referral likely reflects multiple factors: limited patient awareness that psychological support is part of cancer care, the stigma and self‐stigma surrounding mental health, difficulty recognising symptoms of distress, and service‐level barriers including geographical disparity [16, 24, 25, 26]. The consistency of the under‐representation view suggests services are operating at ceiling and cannot absorb further referrals, rather than that the cancer population is not distressed, a mismatch that leaves many patients with unmet psychological needs. This rate reflects access to specialist (Level 3–4) services only. Lower‐intensity support at NICE Levels 1–2, delivered by non‐psychological specialist cancer staff, and provision by third‐sector organisations meet part of this need outside specialist services; the 8.4% therefore indexes demand reaching specialist psychological provision rather than implying that other patients receive no form of psychological support.

How this under‐referral is distributed within the cancer population is a question this service‐level survey cannot answer. Because access depended largely on clinicians recognising distress and on patients self‐advocating, those less able to navigate services may be less likely to be referred; evidence from other settings indicates that access to psychosocial care is socially patterned by socioeconomic position and ethnicity [27, 28, 29]. We collected no patient‐level demographic data and cannot test whether the 8.4% referral rate is evenly distributed—an important priority for future research linking referral to deprivation and ethnicity.

A second observation concerns psychological leadership. The small number of services without a dedicated senior leadership post (band 8b or above) tended to have longer waits (median routine 4 vs. 3 months; urgent 30 vs. 10 days). This finding should be interpreted cautiously, as the comparison is observational and only four services lacked such a post. The difference may also reflect other characteristics of these services rather than an effect of leadership itself. With that caveat, senior posts, typically banded at 8b and above depending on service size, provide embedded psychological leadership and supervision to the wider team and help ensure psychologically informed care across all levels of a cancer service, including indirect work such as Level 2 skills training and supervision [30, 31]. Leadership alone, however, cannot deliver the volume of specialist intervention required. The near‐universal presence of Level 4 staff alongside Level 3 provision in only 40% of services inverts the stepped‐care model, in which the greater share of specialist psychological need is at Level 3. Where sufficient senior leadership is already in place, through a band 8b post, or consultant‐level 8c/8d leadership in teams of three or more staff, psycho‐oncology staffing should, arguably, be balanced to meet need across both Levels 3 and 4.

Because the survey included only services with existing psychological provision, the gaps identified are likely to underestimate the national picture. Services with little or no provision were not represented and may face even greater shortfalls. Taken together, these findings support the case for specialist, embedded psychological support, supported by appropriate leadership, as a core component of comprehensive cancer care [9, 30].

Referral governance varied between services. Any member of the hospital multidisciplinary team could typically refer, but only around a third accepted GP (primary care) referrals and about a tenth accepted self‐referrals or referrals for family members or carers [9, 32]. These findings need to be considered in the context of how cancer pathways are commissioned: primary care is not typically commissioned to refer directly into hospital‐based specialist cancer psychology, so the proportion of services accepting GP referrals may in fact be comparatively high. Self‐referral likewise carries trade‐offs. It may improve access and autonomy, but raises questions about triage, demand management and whether those who self‐refer are representative of those with greatest need. Wider self‐referral is therefore not necessarily preferable. Nevertheless, variation in who can refer, who can be seen and for what remains relevant to the equity intended by the framework, and warrants consideration when referral pathways are defined.

A further area to note is the lack of standardised outcome measurement. Although the PHQ and GAD were widely used, services frequently used different instruments and versions, and only a minority used a validated QoL measure. This variability in outcome measures used limits inter‐service comparability and evaluation of effectiveness. The instruments in use were, moreover, designed to screen for anxiety and depression or to assess generic health‐related quality of life, and reviews of the available measures underscore how heterogeneous and inconsistently applied they remain [33, 34]. No national standard specifies a single outcome measure for psycho‐oncology, so this variation is unsurprising and not necessarily problematic; standardisation brings comparability but reduces local flexibility, and healthcare systems differ in how far they mandate it. Even so, a psychometrically robust, clinically usable measure of psychological wellbeing developed specifically for people living with and beyond cancer would be a useful addition, allowing the impact of specialist input to be captured and compared more consistently.

## 5 Implications (Clinical and Research)

### 5.1 Clinical Implications

The findings point to an urgent need to expand and rebalance the specialist psycho‐oncology workforce, with senior Level 4 leadership (band 8b and above) matched by sufficient Level 3 and 4 capacity to meet need across both tiers. For cancer services with little or no provision, this means establishing specialist, embedded psychological support as a core component of comprehensive cancer care, with appropriate leadership to sustain it. Because referral is clinician‐led in most services, recognition of distress across cancer teams is important; wider awareness, supported by Level 2 skills training and supervision, may help promote timely and appropriate referral. Psycho‐oncology services should also routinely audit referral patterns to identify potential areas of inequity. Psychological specialists are well placed to work alongside people with lived experience to review referral patterns, routes into services and barriers to accessing specialist psychological support. Adequate psychological support has been associated with reduced downstream healthcare use [11, 25], although this survey cannot assess cost‐effectiveness directly. While these findings are framed within the English and Scottish NHS, the principles of matching specialist leadership with capacity across tiers and improving recognition and referral are likely to apply to other publicly funded cancer systems.

### 5.2 Research Implications

Three priorities follow. First, the survey should be repeated with a larger UK‐wide sample, including services in Northern Ireland and Wales, to provide a more complete national picture. A standardised minimum dataset covering waiting times, staffing, activity and outcomes, ideally coordinated regionally and collected through digital tools to reduce administrative burden [35], would also allow more consistent monitoring and comparison of psycho‐oncology provision over time. Second, there would be value in developing and validating a clinically usable, cancer‐specific measure of psychological wellbeing, building on existing instruments and the current literature [33, 34], to allow the impact of specialist input to be evaluated more consistently across services. Third, the inequities in access suggested here warrant dedicated investigation. Research linking referral to psycho‐oncology services with deprivation, ethnicity and other markers of disadvantage is needed to quantify who is missing from these gatekept pathways and to test approaches that widen and equalise access.

## 6 Limitations

### 6.1 Study Limitations

The data represent a cross‐sectional snapshot of 2022/2023 and may not capture within‐year variation or staffing changes such as long‐term sickness. The survey deliberately focused on services with established specialist psychology provision; services with little or no provision could not be surveyed, as the questionnaire items would not have been applicable. Participation was voluntary, so the responding services may represent a self‐selected subset of psychology‐led services. Despite UK‐wide distribution, no responses were received from Wales or Northern Ireland, so the findings describe England and Scotland only. Missing data across several items, including new diagnoses, WTE staffing and activity, reduce the completeness of some calculations and may bias estimates. The number of contributing services is therefore reported for each metric, and the waiting‐time comparison by leadership rests on very small numbers (four services without a dedicated senior leadership post). Self‐reported denominators (e.g., trust‐level diagnoses) varied in whether non‐melanoma skin cancers were included, and variability in local recording (such as how cancelled appointments were counted) may affect comparability.

## 7 Conclusion

This survey of psycho‐oncology services in England and Scotland reveals a substantial mismatch between the psychological needs of people affected by cancer and the capacity of UK specialist psycho‐oncology services, even among services that are already psychology‐led. Fewer than one in 10 patients are referred, those seen face long waits, access is inequitable and referral governance inconsistent. Specialist leadership matters; the few services without a dedicated senior leadership post tended to wait longer, but leadership must be underpinned by balanced additional staffing across Levels 3 and 4 to translate into timely, sufficient care. Delivering equitable and effective psychological care across the cancer pathway will require investment in and rebalancing of the workforce, more consistent referral routes, development of a cancer‐specific psychological wellbeing measure, and specialist provision supported by appropriate leadership where this is currently absent. In setting out what well‐resourced services look like, these findings also provide a benchmark for making that case. They also show how a national policy framework is being implemented in practice, providing evidence that may be relevant to other publicly funded cancer systems facing similar pressures.

## Author Contributions

**Anna Lagerdahl:** conceptualisation, methodology, investigation, formal analysis, supervision, project administration, writing – original draft, writing – review and editing. **Vanessa Bútorová:** formal analysis, data curation, writing – review and editing. Both authors take responsibility for the integrity of the work and its published account, the second author's contribution comprised analysis and the original write‐up of the survey dataset. All authors reviewed and approved the final version prior to submission.

## Funding

The authors have nothing to report.

## Conflicts of Interest

The authors declare no conflicts of interest.

## Data Availability

The data that support the findings of this study are available on request from the corresponding author. The data are not publicly available due to privacy or ethical restrictions.
